# Protective factors against oxidative stress in COPD: focus on Nrf2-dependent antioxidant gene expression

**DOI:** 10.3389/fmed.2025.1492256

**Published:** 2025-05-02

**Authors:** Eva Kominkova, Martin Petrek, Zdenka Navratilova

**Affiliations:** Department of Pathological Physiology, Faculty of Medicine and Dentistry, Palacky University Olomouc, Olomouc, Czechia

**Keywords:** chronic obstructive pulmonary disease, antioxidants, antioxidant gene expression, oxidative stress, Nrf2/ARE signaling pathway, antioxidant treatment

## Abstract

Chronic obstructive pulmonary disease (COPD) continues to be the world’s primary cause of morbidity and mortality. The main mechanism driving the pathogenesis of COPD is oxidative stress. Antioxidant genes, regulated by the Nrf2/ARE signaling pathway, play a protective role against oxidative stress. Unfortunately, this pathway appears to be dysregulated in COPD, leading to decreased expression of antioxidant genes and persistent oxidative stress. We reviewed numerous studies measuring the expression of antioxidant genes in COPD. We also focused on developments in methods used to study gene expression in COPD over time, along with measuring antioxidant gene expression in various cell types, and the potential use of antioxidant gene expression as a predictor of COPD progression. And last but not least we discussed the association of cigarette smoke exposure with antioxidant gene expression together with antioxidant treatment in COPD. Understanding the altered expression of antioxidant genes in COPD could help in treating COPD, as well as predicting its progression.

## Introduction

One of the leading causes of morbidity and mortality worldwide still remains chronic obstructive pulmonary disease (COPD). Moreover, its prevalence and burden are expected to rise over the following decades due to a combination of continuing exposure to COPD risk factors and global population aging. The current definition of COPD according to the GOLD 2025 report is stated as follows: COPD is a heterogeneous lung condition characterized by chronic respiratory symptoms (dyspnea, cough, sputum production and/or exacerbations) due to abnormalities of the airways (bronchitis, bronchiolitis) and/or alveoli (emphysema) that cause persistent, often progressive airflow obstruction (1). The COPD definition’s revision was proposed by Celli et al. (2). Regarding etiology, it is widely accepted that inflammatory damage to the lung is necessary for the development of COPD. This is typically associated with exposure to cigarette smoke in high-income countries and exposure to biofuel combustion in middle-and low-income countries. However, since some people develop COPD despite having no documented environmental exposures and among smokers, only a minority develop COPD, the involvement of genetic susceptibility is suggested. Alpha-1 antitrypsin deficiency is the best-defined genetic factor (3, 4).

Numerous factors influence the prognosis of COPD. The ones that not only worsen the prognosis but also increase burden and mortality are COPD exacerbations (1). That is why Hurst and colleagues systematically reviewed prognostic risk factors for moderate to severe exacerbations in patients with COPD. They proposed that a history of exacerbations in the preceding year is the most accurate predictor of future exacerbations, along with disease severity, bronchodilator reversibility, and comorbidities (5). The most influential comorbidities associated with a poor prognosis include anxiety and depression, dyspepsia, cardiovascular and respiratory issues. The problem is that anxiety and depression are underdiagnosed and untreated (5–7). Other indicators of a poor prognosis are short distance walked in 6 min, weight loss, and malnutrition (1). Persistent smoking is also known to be associated with a poor prognosis. Interestingly, patients who started smoking at an early age (smoking initiation ≤ 24 years old) have been shown to have a worse prognosis than those who began smoking later in life (8). The current gold standard for diagnosing and assessing the severity of COPD is spirometry (9). In COPD patients with forced expiratory volume in 1 s/forced vital capacity (FEV1/FVC) < 0.7, the severity of airflow obstruction is divided into GOLD grades ranging from mild (GOLD 1), moderate (GOLD 2), severe (GOLD 3) to very severe (GOLD 4) based on post-bronchodilator FEV1 (1). Formerly, GOLD 0, defined by the presence of smoking, chronic cough, and sputum production with FEV1/FVC > 0.7, i.e., with normal spirometry, was also embodied in this grading. However, this grade has been dropped over time as not all of these individuals developed COPD. Given how extensive under and misdiagnosis nowadays lead to patients receiving no or incorrect treatment, this may not have been the wisest choice in retrospect (1, 10). The reason for such statement is that when taking into consideration only FEV1/FVC < 0.7, individuals with early-stage disease, having reversible or delayable pathologic changes, might be omitted (11). As appropriate and earlier diagnosis of COPD can have a very significant public-health impact, it is crucial to define a variety of clinical (bio)markers that embrace the term “pre-COPD” to identify individuals who have normal spirometry at present yet are at increased risk of developing COPD in the future. Apart from these diagnostic biomarkers, biomarkers of COPD progression to distinguish among subsequent COPD stages would also be useful (1, 12). Since spirometry has its limitations in diagnosing COPD (technique-dependent, nonspecific administration by a trained healthcare professional required), new alternative diagnostic tests are also needed. Such a fast, reliable, and precise alternative for spirometry was tested in a study by Talker et al. They used interpretable machine learning and TidalSense’s N-Tidal™ capnometer to diagnose COPD and evaluate its severity with promising results (9).

The main mechanism driving the pathogenesis of COPD is oxidative stress, which is further increased during exacerbations as oxidants are induced by cigarette smoke and other inhaled toxic particles. Oxidative stress comprises reactive molecules and free radicals derived from molecular oxygen, altogether known as reactive oxygen species (ROS) (1, 12, 13). These include, e.g., hydroxyl radicals, hydrogen peroxide, peroxynitrite, nitrogen dioxide, and others (14, 15). The immune cells responsible for the release of ROS, but also other pro-inflammatory factors, e.g., neutrophil elastase and matrix metalloproteinases (MMPs) are neutrophils and macrophages (16, 17). Cigarette smoke exposes lungs to exogenous ROS when 5 × 10^14^ free radicals are thought to be released with each “puff.” Numerous of these free radicals have a half-life that allows them to enter the lower respiratory tract (15, 18). This results in an imbalance between oxidants and antioxidants in the lungs leading to excessive oxidative stress which amplifies airway inflammation and alters cellular metabolism causing the protease-antiprotease imbalance, along with activation of the DNA damage response and induction of cellular senescence (19–21). All these cause the loss of elasticity and narrowing of the airways, making it difficult for people with COPD to breathe (17). However, oxidative stress perseveres even in ex-smokers, indicating that oxidative stress occurs also endogenously (22). These endogenous ROS are produced mainly by malfunctioning or damaged mitochondria. Such ROS-induced oxidative stress damage has been demonstrated to be countered by antioxidants (13). However, it seems that in stable COPD and mainly during exacerbations there is a decrease in their production and/or function (23). This indicates that antioxidants should be efficient in COPD treatment, particularly in preventing the disease progression and exacerbations (24).

In terms of COPD treatment, bronchodilators are suggested as the first-line treatment in all stages of COPD severity by the GOLD 2025 guidelines, typically in conjunction with inhaled corticosteroids (ICS). We distinguish between short-acting and long-acting bronchodilators. The short-acting ones are short-acting beta2-agonists (SABAs) along with short-acting muscarinic antagonists (SAMAs). Whereas the long-acting ones are long-acting beta2-agonists (LABAs) and long-acting muscarinic antagonists (LAMAs). Oral corticosteroids (OCS) can also be used (1, 25). Even though several studies showed that steroid treatment is rather ineffective in improving lung function and reducing airway inflammation in patients with COPD, nowadays used triple therapy (LABA + LAMA + ICS) has been shown to improve lung function and reduce exacerbations more efficiently than either component alone (1, 26, 27). Despite these significant therapeutic benefits, these drugs have some limitations (27). Corticosteroids have several side effects including serious infectious complications which were reported already in 1989 by Wiest et al. (28). Even though long-acting bronchodilators typically have modest side effects, patients with COPD and a history of heart failure or cardiovascular disease may be more vulnerable to uncommon, severe side effects, such as cardiac arrhythmias (29). Moreover, the concurrent use of long-acting bronchodilators in COPD is also linked to the risk of adverse cardiovascular events. This is because long-acting anticholinergics are assumed to suppress parasympathetic control, and LABAs stimulate sympathetic tone, which might lead to tachyarrhythmia and coronary insufficiency (30). Since the use of bronchodilators and corticosteroids is still critically essential for the management of COPD, the development of alternative, more effective, and safer therapies is needed. As recent studies showed positive effects of specific novel antioxidants, antioxidant treatment will be given more attention later in a separate paragraph (27, 31). Besides pharmacological treatment, we must not forget non-pharmacologically focused strategies for treating COPD. These include for example smoking cessation, pulmonary rehabilitation, oxygen therapy, ventilatory support, nutrition, physical activity, and others (1, 25).

This paper aims to review the role of antioxidants against oxidative stress in COPD, with a focus on antioxidant gene expression. Antioxidant treatment receives attention as well.

## Genes involved in oxidative stress response in COPD

As oxidative stress is the main mechanism driving the pathogenesis of COPD, genes that take part in the oxidative stress response also have a crucial role in the pathogenesis of COPD. The key role plays the Nuclear factor erythroid 2-related factor 2 (Nrf2) transcription factor, which is essential in protecting lung cells from oxidative stress by upregulating antioxidant genes in response to tobacco smoke exposure in COPD patients (32). Its importance was unmasked already in 2004 by Rangasamy et al. as the disruption of the Nrf2 gene in mice led to earlier-onset and more extensive emphysema in response to cigarette smoke compared to Nrf2 wild-type littermates, which signifies that mice lacking Nrf2 are more susceptible to CS-induced emphysema/oxidative stress (33). Iizuka and colleagues confirmed this discovery a year later (34). The same results were observed in another study, which focused on examining the susceptibility of Nrf2-knockout mice to elastase-induced emphysema (35). As the protective effect of Nrf2 against oxidative stress lies in the Nrf2-dependent induction of antioxidants, all studies, therefore, focused also on measuring the expression of several antioxidant genes (e.g., HO-1, NQO1, etc.). The expression of these antioxidant genes was upregulated in the lungs of Nrf2 wild-type mice, but not in the lungs of Nrf2 knock-out mice (33–35). Silencing RNA experiments *in vitro* in THP-1 cells performed by Goven et al. in 2008 verified that the loss of Nrf2 results in a decrease of Nrf2-regulated antioxidant gene mRNA expression, namely HO-1, NQO1, and GPX2 (36).

**Figure 1:**
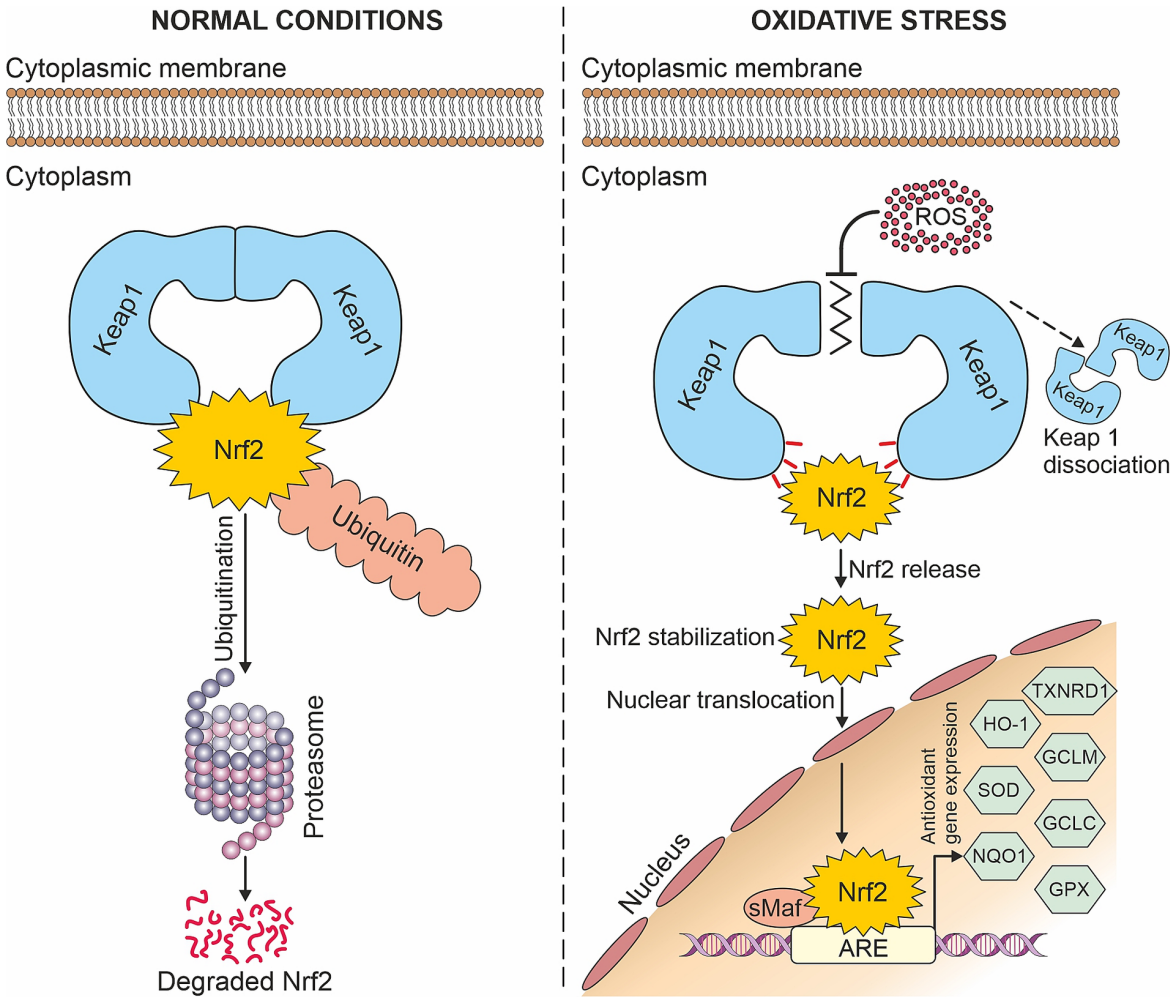
Nrf2/ARE signaling pathway: Under normal conditions, Nrf2 is degraded in the proteasome, whereas in the presence of oxidative stress, it is translocated into the nucleus where it induces antioxidant gene expression.

The Nrf2-dependent induction of antioxidants is regulated by the Nrf2/ARE signaling pathway. Under normal conditions, low levels of Nrf2 are maintained since Nrf2 binds to its cytoplasmic inhibitor Kelch-like ECH-associated protein 1 (Keap1) and is subsequently proteasomally degraded. However, in the presence of oxidative stress which modifies the inhibitor Keap1, Nrf2 is released, stabilized, and translocated into the nucleus. This allows Nrf2 to form heterodimers with small Maf proteins, which bind to antioxidant response elements (AREs) and induce the expression of antioxidant genes (see Figure 1). These include for example superoxide dismutases (SODs), heme oxygenase-1 (HO-1), glutamate-cysteine ligase catalytic (GCLC) and modulator (GCLM) subunits, NADP(H): quinone oxidoreductase-1 (NQO1), glutathione peroxidases (GPXs), thioredoxin reductase-1 (TXNRD1) and others (21, 37–39).

Even though Nrf2 is the key player in antioxidant protection, other transcription factors and negative regulators participate in the production of antioxidants. It was found that the transcription factor BTB domain and CNC homolog 1 (Bach1) represses ARE activity by competing with NRF2 for ARE binding, thereby negatively controlling the transcription of genes such as HO-1 or NQO1 (40, 41). On the other hand, DJ-1 acts as a stabilizer of Nrf2 as it inhibits the Keap1-Nrf2 interaction and therefore proteasomal degradation of Nrf2 (42). Amatullah et al. demonstrated that the loss of DJ-1 leads to increased oxidative stress (43). Another study found that the loss of DJ-1 results in a decreased expression of NQO1. This is because DJ-1 is required for the activity of Nrf2. If it is not present, Nrf2 is unstable, which results in decreased transcriptional responses (39).

Nevertheless, this pathway seems to be dysregulated in COPD. In 2008 a study by Goven et al. showed altered Nrf2/Keap1-Bach1 equilibrium in pulmonary emphysema (36). Moreover, according to Li et al. there is strong evidence of downstream dysregulation of the Nrf2 signaling pathway in alveolar macrophages (AM) from COPD patients (44). Unfortunately, little is known about the molecular mechanism behind COPD patients’ decrease in Nrf2 signaling (21). It might be caused by a reduction in the stabilizing protein DJ-1 and/or increased Keap1 levels leading to increased NRF2 proteasomal degradation and therefore decreased expression of antioxidant genes resulting in persistent oxidative stress (41, 45).

As stated by Bahmed et al. restoring such an impaired antioxidant defense system could be possible by DJ-1 overproduction (46). It could also be possible by deleting Keap1 as in 2009 experiments on a novel conditional knockout mouse model with genetically deleted Keap1 in airway epithelial cells (Clara cells) showed that this deletion increased expression of Nrf2 dependent genes (e.g., NQO1, GCLM, GCLC, TXNRD1, GPX2). Such increased Nrf2 activation reduced oxidative stress (47).

As activation of the Nrf2/ARE antioxidant pathway is believed to be an efficient therapeutic strategy for redox-related lung diseases, it will be discussed later in a paragraph titled Antioxidant treatment in COPD (42).

## Relative expression of the Nrf2/ARE pathway genes in COPD

We are interested in this topic because of the rapidly increasing prominence of the use of biomarkers in COPD (1). Numerous studies have measured the Nrf2-dependent gene expression but with rather varying results. An overview of these studies is shown in Supplementary Table 1. These inconsistent results may be due to the variability in the used biological material, methodology, control samples, and others. It is also important to point out that each study has its limitations, whether it is a small sample size which reduces the statistical power of the study (32, 36, 48–51), heterogeneity of COPD patients enrolled in the studies such as grouping of subjects with different GOLD stages into one group, as in a study by Pierrou et al. where COPD stages 2–4 were grouped together (48, 52–54) or not taking into account the COPD severity at all (32, 44, 49, 55–59) however, these studies focus mainly on the comparison of smoking status and COPD. In surgical tissue studies a frequent problem is the nonavailability of healthy controls, as control samples are often used paracancerous tissues from lung cancer patients, which might influence the results (36, 44, 48, 50, 51, 53, 55, 56, 59–63). Moreover, in the case of longitudinal studies, large amounts of data can go missing between the baseline and follow-up period (64). In succeeding paragraphs, we will discuss these issues and more.

## Progression of methods used to study COPD gene expression

This section will focus on reviewing the developments in methods used to study gene expression in COPD over time. The basal method to measure the expression at the protein level is probably immunohistochemical analysis (81, 99, 100). This analysis is based on staining various tissues and counting the number of gene-positive nuclei. The problem is that commonly used are lung tissue samples often obtained from patients undergoing resection for lung tumor, which may influence the expression (56, 60, 61). Nowadays, immunohistochemical analysis is rather used as a complementary analysis either to western blotting (48, 50, 51, 59, 65) or RT-qPCR, which is undoubtedly the most commonly used method to measure gene expression (32, 36, 44, 49, 55, 62, 64, 66, 67, 97, 98).

All mentioned methods serve to measure gene expression more or less well; however, they are suitable for measuring only a few genes. Luckily with the development of more advanced technology, a bigger amount of data can be analyzed. As today’s standard is considered microarray analysis, most often using Affymetrix arrays (54, 68, 69). Such obtained microarray data are frequently confirmed by RT-qPCR and deposited in the Gene Expression Omnibus (GEO) website so other studies can use these microarray datasets for further analysis. For instance, Lin et al. identified differentially expressed genes (e.g., NQO1 among them) between COPD smokers and non-smokers in airway epithelium. Such genes could be used as potential biomarkers for the diagnosis and treatment of COPD, all thanks to these available datasets (58). Studies of this kind often do not focus only on measuring gene expression but also on analyzing pathways (gene sets), as the research by Pierrou et al., 2007. In addition to the assessment of global gene expression, they also performed gene set enrichment analysis (GSEA), which revealed that pathways involved in oxidant/antioxidant responses were among the most differentially expressed ones in smokers, with further differences seen in COPD patients (52).

Over the past decade, RNA sequencing has become an indispensable tool for transcriptome-wide analysis of differential gene expression, serving as an alluring substitute offering many advantages over conventional microarray platforms (70, 71). Ghosh et al. with the help of RNA sequencing identified a set of genes that overlap between multiple clinically relevant phenotypes and demonstrated that the overlapping genes are also associated with COPD progression (72). With the development of next-generation sequencing technologies, RNA sequencing developed as well. Currently, it may be used to analyze even single-cell gene expression. The study by Sauler et al. used single-cell RNA sequencing to characterize the COPD alveolar niche and, among other things, to find several differentially expressed genes (DEGs) encoding antioxidants (73). Whereas An et al. used single-cell transcriptome sequencing not only to compare cell subsets in COPD and controls (74). As the cost of single-cell sequencing is relatively expensive at present, the sample size studied is small. There is a need for more COPD-related single-cell sequencing data available so the results can be validated by performing more in-depth data analysis.

Regarding the study type, studies focusing on measuring only a few genes on a small sample size have been sidelined lately. That is why a promising direction might be to focus on performing desirable longitudinal studies. A good example is an already mentioned article written by Fratta Pasini et al. dealing with Nrf2/ARE gene expression and lung function in COPD patients over time (64). Desired are also studies comprising of a huge number of patients involved and a lot of genes analyzed. These studies often pool COPD patient samples from large longitudinal observational studies. A great example is the ECLIPSE study (Evaluation of COPD Longitudinally to Identify Predictive Surrogate Endpoints) which is a large observational study of COPD patients and controls conducted at 46 centers in 12 countries aimed at defining COPD phenotypes and identifying biomarkers and genetic parameters that help to predict disease progression. In three years, they managed to recruit 2083 COPD subjects, 332 control smokers and 237 non-smoking control subjects. Apart from the baseline visit, the subjects of the study were followed up at a total of seven visits at 3 months, 6 months, and every 6 months thereafter for 3 years (75, 76). This cohort, along with subjects from additional COPD case–control studies, was used for instance by Qiu and colleagues, 2011 in a GWAS analysis studying the genetics of sputum gene expression in COPD (77). Another study using the ECLIPSE cohort performed GWAS of circulating COPD biomarkers, finding some novel loci affecting the levels of plasma protein biomarkers (78). The biomarker results of the ECLIPSE study were reviewed by Faner et al. (79). These so-called Genome-wide association studies (GWAS) examine single nucleotide polymorphisms (SNPs) across the genome enabling not only the identification of genes associated with a particular disease. Nowadays the sample sizes comprise hundreds of thousands of individuals. The downside of these studies is that they are inherently very expensive, so conducting such research is often a matter of funding.

The well-known COPDGene Study with over 10,000 patients involved and more than 425 publications resulting from the research of COPDGene investigators is one of the largest studies ever to investigate the underlying genetic factors of COPD and is as well designed longitudinally. At the moment, all participants enrolled in Phase 1 are being invited for a third study visit which is approximately ten years after their first visit, and participants enrolled in Phase 2 are currently being invited for their second study visit approximately five years after their first visit. Already mentioned articles studying gene expression and pathways in COPD (54, 72) are a part of the COPDGene Study. More information about the COPDGene Study and a list of publications can be found at: https://www.copdgene.org.

## Measuring antioxidant gene expression in various cell types in COPD

As the primary site of the disease in COPD is the lung, most often the antioxidant gene expression is measured in lung tissue (36, 44, 48, 49, 51, 53, 55–57, 59, 60, 65, 73, 80, 97) commonly the interest is shifted mainly to alveolar macrophages because they play a crucial role in the pathogenesis of COPD (36, 44, 53, 57, 60, 73) but also bronchial and airway epithelium (44, 53, 57, 60, 65, 73). Unfortunately, these studies typically have a small sample size because of the invasiveness of obtaining lung tissue samples. The results might be influenced by the presence of cancer because the subjects used are often undergoing lung resection for lung cancer. A less invasive option how to obtain alveolar macrophages, bronchial and airway epithelium samples is bronchoscopy (32, 53, 68, 69, 81). However, subject recruitment is also quite difficult as bronchoscopy is not commonly used to diagnose COPD, unlike sarcoidosis, moreover, it is not a pleasant procedure.

While the lung is the site of primary exposure to tobacco smoke, COPDGene investigators have confirmed the systemic symptoms of COPD by identifying numerous blood biomarkers even after adjusting for smoking. Thus, blood samples might be ideal for assessing these systemic effects and gene expression in COPD. Moreover, blood collection has several advantages over lung sampling: it is not so invasive and time-consuming, thus repeated sampling to monitor disease progression is possible, making blood practical in large research cohorts and biomarker screening where a large number of samples is needed (54, 82).

From collected blood can be isolated peripheral blood mononuclear cells (PBMCs) which are precursors of the alveolar macrophages (32, 54, 64, 66, 83) or peripheral blood leucocytes (PBLs) (84) which can be subsequently used for the analysis of gene expression and plenty of other experiments. Data obtained from such studies can be further analyzed. An and colleagues reanalyzed the scRNA-seq data of peripheral blood mononuclear cells of COPD patients downloaded from the Gene Expression Omnibus (GEO) database and besides several experiments, they performed a correlation analysis between the expression and immune infiltration abundance of COPD immune-related genes and found high positive correlation of HO-1 in macrophages (74).

Even though PBMC gene expression has a big potential to be used as a biomarker of COPD (54), not many studies have focused on measuring the Nrf2-target gene expression in the blood of COPD patients so far. Most likely, this issue has received the greatest attention from Fratta Pasini et al. They performed two studies, in the first one they found increased mRNA and protein expression of Nrf2 and HO-1 in PBMCs from mild–moderate (ex-smoker) COPD patients (66). In the second study, they confirmed these results, as the mRNA expression of Nrf2, HO-1, and in addition, GCLC was increased at baseline (= mild–moderate COPD) compared to controls (64). Unfortunately, these results are not in line with those from a study by Sidhaye et al. where neither of the studied genes (Nrf2, HO-1, NQ01, Keap1, and others) showed statistically increased expression. However, this may be due to the fact that they did not use healthy controls as Fratta Pasini et al. but former smokers with COPD (32, 64). A study by Bahr et al. involved a really big sample size (*n* = 211) and all COPD GOLD stages, which makes it one of the largest studies of PBMC microarray gene expression profiling for COPD in humans. They found a negative correlation of Bach1, SOD1, VDR, and FOXO1 with FEV1 (54). More studies measuring the Nrf2-target gene expression in the blood of COPD patients are needed.

It is important to note that presumably, increased Nrf2 activity is a response to local exposure, which explains why peripheral blood cells may not exhibit comparable alterations. Thus, substituting lung tissue for PBMCs must be well considered as studied expression changes might be compartmentalized to the lung (32). As mentioned earlier, many other factors can influence the expression, so results may vary even when measuring the expression in the same cell type. At variance with studies measuring the Nrf2 target gene expression in PBMCs, those measuring it in the lung found decreased expression of several Nrf2 target genes (36, 53, 56, 60, 81, 97). On the other hand, numerous studies measured increased expression (48, 51, 52, 57, 58, 69, 73). More studies measuring the Nrf2-target gene expression in blood and lung tissue from the same subjects, as was done by Sidhaye et al. are required to clarify these dissimilarities.

Adair-Kirk and colleagues discovered that Nrf2-regulated genes are also upregulated in Clara cells, which are non-ciliated secretory cells present within the bronchiolar epithelium of the mammalian lung (85). This is not surprising as Sidhaye et al. showed that bronchial epithelium is the most responsive tissue for transcriptional activation of Nrf2 target genes (32). Clara cells are an important source of antioxidant/detoxification gene expression in response to cigarette smoke exposure via the Nrf2 transcription factor. Therefore, altered Clara cell function is likely to be a significant factor contributing to lung function decline (85, 86). Fortunately, Clara cell function, and thus lung function could be protected by increased Nrf2 activation resulting in reduced oxidative stress. As shown in already mentioned experiments by Blake et al. on a novel conditional knockout mouse model with genetically deleted Keap1 in Clara cells, where such deletion led to increased expression of Nrf2 dependent genes, e.g., NQO1, GCLM, GCLC, TXNRD1, GPX2 (47).

## Antioxidant response gene expression as a predictor of COPD progression

In 2007 a longitudinal cohort study showed that increased serum CRP is a strong long-term predictor of COPD hospitalization and death, in other words of COPD progression (87). Later it was revealed that combinations of biomarkers (e.g., CRP, fibrinogen, SP-D) improve the predictive value (88). Several studies indicate that antioxidant-related genes might also be used for this purpose.

It has been hypothesized that decreases in the expression of oxidant response genes may reflect a loss of defensive capabilities against infection as the disease becomes more severe. Pierrou et al. found that the expression of a number of genes involved in oxidant stress responses (namely GPX2, GCLM, GCLC, and others) was increased in healthy smokers compared with nonsmokers. This phenomenon was even stronger when compared with COPD stage 0 and the strongest in stage 1. Whereas in more severe COPD (stage 2–4) the mean expression fell to the same levels as in stage 0 (52). This hypothesis which practically means that the increase in antioxidant defenses is typical for the early stages of the disease and with the severity of the disease antioxidant defenses gradually fail is supported in a study by Fratta Pasini et al. In this longitudinal study, they determined that at baseline the expression of Nrf2 and Nrf2-related genes (heme oxygenase (HO)-1 and glutamate-cysteine ligase catalytic (GCLC) subunit) in PBMCS was significantly increased in COPD compared to no-COPD subjects, whereas at the follow-up (49.7 ± 6.9 months) the expression was significantly decreased in COPD which worsened over that time, while no changes were observed in no-COPD subjects. Moreover, the percent variation (*Δ*) of FEV_1_ detected after the follow-up in COPD patients was directly correlated with ΔNrf2 (*r* = 0.826, *p* < 0.001), ΔHO-1 (*r* = 0.820, *p* < 0.001), and ΔGCLC (*r* = 0.840, *p* < 0.001), which means they can be taken as significant predictors of ΔFEV_1_ (64).

**Figure 2:**
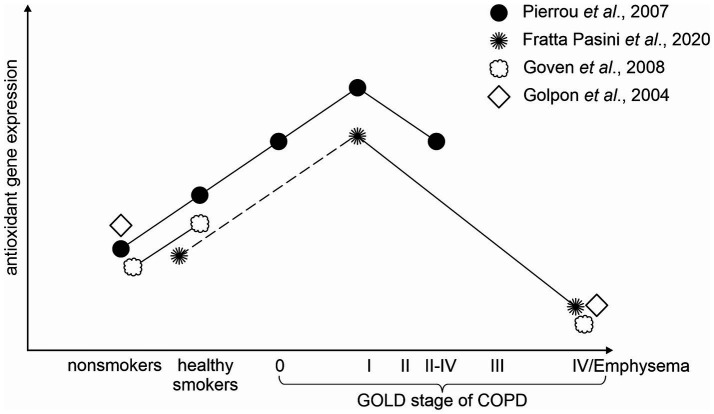
Visual representation of the hypothesis that the antioxidant gene expression decreases with COPD severity.

Even though not many studies distinguish among different stages of COPD when analyzing the gene expression, we were able to find some other studies supporting this hypothesis (see Figure 2). A study by Goven et al. found a decreased expression of HO-1, GPX2, and NQO1 in severe emphysema (36). Another study reported that severely emphysematous lung tissue is characterized by a decline in GPX3 gene expression (80). Wang with his colleagues showed a decreased expression of FOXO3A as airway obstruction progressed (63). Accordingly, the expression of NOX4, which is a major source of oxidative stress, was found to be increased with COPD severity (65).

Recently Zhang and colleagues identified numerous differentially expressed genes (DEGs) candidates of COPD progression (with antioxidant-related genes among them) in response to smoking. Furthermore, they created practical and efficient discriminant models that can precisely predict COPD progression (89).

## Association of cigarette smoke exposure with antioxidant gene expression

It is alarming that about 40% of COPD patients are current smokers despite knowing that persisting smoking has a high correlation with the development of emphysema, disease progression, and poor prognosis (1, 90). In a recent study, it has been well established that smoking affects gene expression patterns (89). The question we focused on is how smoking affects the Nrf2 target gene expression in smokers with and without COPD.

In 2003 Hackett et al. performed a study based on a hypothesis that oxidants are the major mechanism of smoking-induced airway damage, therefore smoking should be associated with upregulation of various antioxidant-related genes in the airway epithelium. From 44 antioxidant-related genes, 16 (including GPX2, GPX3, GCLM, GCLC, and TXNRD1) showed a significant upregulation in the airway epithelium of smokers in comparison with nonsmokers (18). This hypothesis was also confirmed by Hübner et al. who demonstrated that Nrf2 is activated in the small airway epithelium of healthy smokers and that Nrf2 modulated genes (e.g., GCLC, GPX2, NQO1, TXNRD1) are highly responsive to cigarette smoking and exhibit a concordant pattern of upregulation in healthy smokers. These findings imply that Nrf2 regulates cellular defenses against smoking in the extremely susceptible small airway epithelial cells (69). Hackett et al. also noted a high inter-individual variability in the expression levels of the upregulated genes, interestingly some smokers had high expression levels of a subset of genes, whereas others exhibited low expression levels of the same gene subset (18). Suggesting that the Nrf2 target gene expression is probably also influenced by genetic factors.

Increased Nrf2 target gene expression was also detected by Fukano et al. while exposing the human lung epithelial cell line A549 to CSC (cigarette smoke condensate), specifically, H0-1 gene expression was increased in a dose-dependent manner (91). In 2007 Pierrou et al. also confirmed that the oxidative gene expression is influenced by cigarette smoke exposure as the expression of antioxidant genes was increased in the bronchial epithelium of healthy smokers, with even a further increase in smokers with COPD in comparison with nonsmokers (52). When contrasting the Nrf2 target gene expression in current and former smokers with COPD, the expression is significantly higher in current smokers compared to former smokers (32).

## Antioxidant treatment in COPD

One of the most encouraging approaches to antioxidant therapy is the usage of Nrf2 activators that activate numerous antioxidant genes (92). The aforementioned study by Sidhaye et al. was the first to assess the Nrf2 levels and activity in different cellular compartments in response to active tobacco use in patients with COPD. They discovered that levels of Nrf2 expression were higher in bronchial epithelial cells and alveolar macrophages compared to nasal epithelium cells and PBMCs. This kind of research is important because knowing the differences in levels of Nrf2 between different compartments could unveil whether specific cellular targeting is required for the therapeutic efficacy of Nrf2 activators (32). Such Nrf2 activators were tested in a study by Li et al. where all used Nrf2 activators (CDDO, GSK7, MMF, Sulforaphane, and C4X_6665) increased NQO1 activity and expression of Nrf2 target genes (NQO1, HO-1, SOD1, and TXNRD1) in COPD alveolar macrophages. The highest potency for inducing antioxidant activity and gene expression had a novel PPI Nrf2 compound C4X_6665 suggesting its big therapeutic potential. This new compound could be used to address Nrf2 pathway dysregulation in alveolar macrophages of COPD patients (44). Apart from these synthetic Nrf2 activators, numerous naturally occurring and plant-derived Nrf2 activators exist (93). For instance, very interesting research was carried out by Qian et al. using a plant that has a long history as a traditional medicine for cough and asthma in China. They discovered that oxidative stress induced by cigarette smoke in COPD rats was inhibited by *Ginkgo biloba L*. seeds through the Nrf2 pathway. Since Nrf2 is a transcription factor that regulates several antioxidants, it has a significant protective effect on lung oxidative airway disease and is likely to become a potential therapeutic target for emphysema prevention and intervention strategies (94). As reviewed by Satpathy *et* Prasad Parida, 2023, recent research has shown that also curcumin (*Curcuma longa L*.) demonstrated antioxidant activity and a protective effect on COPD (95). Other well-known naturally occurring Nrf2 activators are resveratrol from wine, sulforaphane from broccoli, and many others (93).

According to Barnes, 2022 there is a need to study further these activators of Nrf2, along with mitochondria-targeted antioxidants (mt-antioxidants) as already known thiol-based antioxidants, such as N-acetylcysteine have had disappointing clinical effects so far and some Nrf2 activators (sulforaphane and bardoxolone) seem to be nonspecific and toxic (20, 24). As mitochondrial ROS appears to be the main source of oxidative stress in COPD, mentioned mitochondria-targeted antioxidants might be a more promising approach because they are able to cross the mitochondria lipid bilayer and reduce ROS at its source. Mitochondria-targeted antioxidants are based on the structure of ubiquinone, they can be divided into several groups and include for example mitoQ, mito-TEMPO, and SkQ1. Overall, mitochondria-targeted antioxidants showed promising results in experiments *in vitro*, ex vivo, and in pre-clinical animal models of COPD as well. Human clinical trials are currently being in the process (13, 20). Even though there is not sufficient evidence, we cannot exclude dietary antioxidant supplementation (for instance, vitamin C and E), which might also improve lung function in patients with COPD (92).

With increasing evidence suggesting an acceleration of lung aging in COPD followed by the accumulation of senescent cells, a new therapy called senotherapy is being discussed. The underlying problem is that senescent cells fail to repair tissue damage causing disease progression. As cellular senescence is driven by chronic oxidative stress (mitochondrial), it is thought that novel antioxidants discussed above along with others, e.g., anti-FOXO4 may inhibit the development of cellular senescence and thus reduce COPD progression and mortality. Moreover, given that the majority of COPD patients have two or more age-related comorbidities (cardiovascular diseases and others) and that the same pathways of cellular senescence appear to operate in many age-related diseases, it may be possible to treat them all at once (20, 96).

## Conclusion and future directions

Knowing that COPD is the leading cause of morbidity and mortality worldwide, with an expected increase in prevalence and burden, there is an urgent need for a better understanding of its pathogenesis. Numerous uncertainties still remain concerning the dysregulated Nrf2 signaling pathway in COPD patients. A decrease in the stabilizing protein DJ-1 and/or an increase in Keap1 levels may be the reason, which would enhance NRF2 proteasomal degradation and, in turn, decrease the expression of antioxidant genes, resulting in persistent oxidative stress. However, many more antioxidant gene expression studies are required to solve this issue. Concerning the invasiveness of obtaining lung tissue samples for these studies, blood samples from which can be isolated PBMCs might be a better alternative. As PBMC gene expression has also a big potential to be used as a biomarker of COPD, more studies should focus on measuring the Nrf2-target gene expression in the blood of COPD patients. Also, more longitudinal studies and studies distinguishing among different stages of COPD are needed to confirm the hypothesis that the antioxidant gene expression decreases with COPD severity.

Regarding the use of antioxidants in COPD treatment, one of the most promising appears to be the usage of Nrf2 activators. One such Nrf2 activator termed C4X_6665 is believed to be able to address Nrf2 pathway dysregulation in alveolar macrophages of COPD patients. Mitochondria-targeted antioxidants may be even more promising as mitochondrial ROS appears to be the main source of oxidative stress in COPD. It is thought that these novel antioxidants could inhibit the development of cellular senescence and therefore reduce COPD progression and mortality. This new therapy which is currently being discussed is called senotherapy and it could be of great use in the future.
