# Supplementary material for: Protective factors against oxidative stress in COPD: focus on Nrf2-dependent antioxidant gene expression
Source: Front Med (Lausanne). 2025 May 2;12:1492256. doi: 10.3389/fmed.2025.1492256 (PMC12081257; doi:10.3389/fmed.2025.1492256)
Supplement: Supplementary file 1 [file Table_1.docx]

Table 1. Overview of antioxidant expression COPD studies

| Gene | Disease | Control samples | Expression | Biological material | n | Method | References |
| --- | --- | --- | --- | --- | --- | --- | --- |
| Nrf2 | COPD (GOLD 1-2), never smokers, ex-smokers, smokers | No-COPD, never smokers, ex-smokers, smokers | ↑…..↓ | PBMCs | 60…..33 | RT-qPCR | (64) |
|  | Emphysema (GOLD 3-4), smokers, ex-smokers | No-emphysema, lung cancer patients, never smokers, smokers | ↓• | Lung tissue (whole lung homogenates), AM | 10 | Western blot, immunohistochemical and laser scanning confocal analysis | (36) |
|  | COPD (GOLD 1-2), ex-smokers | No-COPD, ex-smokers | ↑/↑• | PBMCs | 30 | RT-qPCR /Western blotting | (66) |
|  | COPD (mainly GOLD 1-2, 4), ex-smokers | No-COPD, lung cancer patients, never smokers, ex-smokers | ↓ | Peripheral lung tissue, pulmonary macrophages | 10 | RT-qPCR | (53) |
|  | COPD (GOLD 0, 1, 2-4), smokers | No-COPD, smokers | ↑ | Epithelial cells obtained by bronchial brushings | 38 | Global gene expression using Affymetrix arrays | (52) |
|  | COPD (GOLD 1-2, 3, 4) | No-COPD, lung cancer patients | ↓• | Lung tissue | 180 | Western blotting | (48) |
|  | COPD, smokers, ex-smokers | No-COPD, lung cancer patients, never smokers, smokers | ⎯/⎯ • | Lung resected tissue (bronchial epithelium, AM) | 12 | RT-qPCR/Immunohistochemistry | (44) |
|  | COPD smokers | COPD ex-smokers | ⎯ | Bronchial epithelium, nasal epithelium, alveolar macrophages, PBMCs | 54 | RT-qPCR | (32) |
| HO-1 | COPD (GOLD 1-2), never smokers, ex-smokers, smokers | No-COPD, never smokers, ex-smokers, smokers | ↑…..↓ | PBMCs | 60…..33 | RT-qPCR | (64) |
|  | Emphysema (GOLD 3-4), smokers, ex-smokers | No-emphysema, lung cancer patients, never smokers, smokers | ↓/↓• | Lung tissue (whole lung homogenates), AM | 10 | RT-qPCR /Western blot, immunohistochemical and laser scanning confocal analysis | (36) |
|  | COPD (GOLD 2-3), smokers, ex-smokers | No-COPD, lung cancer patients, smokers, 1 ex-smoker | ↓• | Peripheral lung, AM | 11 | Immunohistochemistry | (60) |
|  | COPD (GOLD 1-2), ex-smokers | No-COPD, ex-smokers | ↑/↑• | PBMCs | 30 | RT-qPCR /Western blotting | (66) |
|  | COPD (GOLD 2-3), ex-smokers | No-COPD, ex-smokers | ↓• | AM | 13 | Immunostaining | (81) |
|  | COPD (GOLD 0, 1, 2-4), smokers | No-COPD, smokers | ↑ | Epithelial cells obtained by bronchial brushings | 38 | Global gene expression using Affymetrix arrays | (52) |
|  | COPD (GOLD 1-2, 3, 4) | No-COPD, lung cancer patients | ↑• | Lung tissue | 180 | Western blotting | (48) |
|  | COPD, smokers, ex-smokers | No-COPD, lung cancer patients, smokers | ↑ | AM | 8 | RT-qPCR | (44) |
|  | COPD, ex-smokers, smokers | No-COPD, lung cancer patients, never smokers, ex-smokers, smokers | ⎯ | Peripheral lung tissue | 14 | RT-qPCR | (55) |
|  | Advanced COPD, Emphysema, ex-smokers | No-COPD, smokers, ex-smokers | ↑ | Lung tissue: AM | 17 | ScRNAseq, clustering, differential analysis of cells | (73) |
|  | COPD | No-COPD, lung cancer patients | ↑• | Lung tissue | 40 | Western blotting | (51) |
|  | COPD smokers | COPD ex-smokers | ↑/ ⎯ | Bronchial epithelium/nasal epithelium, AM, PBMCs | 54 | RT-qPCR | (32) |
| GCLC | COPD (GOLD 1-2), never smokers, ex-smokers, smokers | No-COPD, never smokers, ex-smokers, smokers | ↑…..↓ | PBMCs | 60…..33 | RT-qPCR | (64) |
|  | COPD (GOLD 0, 1, 2-4), smokers | No-COPD, nonsmokers | ↑ | Epithelial cells obtained by bronchial brushings | 38 | Global gene expression using Affymetrix arrays | (52) |
|  | COPD, smokers | No-COPD, lung cancer patients, never smokers | ↓•/⎯ • | Lung tissue, central bronchial epithelium, AM/Peripheral lung tissue | 22 | Immunohistochemistry | (56) |
|  | COPD, smokers, ex-smokers | No-COPD, smokers, ex-smokers | ↑ | peripheral lung tissue- alveolar epithelial cells | 11 | mRNA in situ hybridization | (57) |
|  | No-COPD, smokers | No-COPD, nonsmokers | ↑/↑ | Small airway epithelium/AM | 30 | Microarray analysis, with RT-qPCR confirmation | (68) |
|  | No-COPD, smokers | No-COPD, nonsmokers | ↑ | Small airway epithelium | 45 | Microarray analysis, with RT-qPCR confirmation | (69) |
|  | COPD, smokers, ex-smokers | No-COPD, smokers, ex-smokers, never smokers | ↓ | Lung tissue | 58 | RT-qPCR | (97) |
| GCLM | COPD (GOLD 0, 1, 2-4), smokers | No-COPD, nonsmokers | ↑ | Epithelial cells obtained by bronchial brushings | 38 | Global gene expression using Affymetrix arrays | (52) |
|  | COPD, smokers | No-COPD, lung cancer patients, never smokers | ↓•/⎯ • | Lung tissue, central bronchial epithelium, AM/Peripheral lung tissue | 22 | Immunohistochemistry | (56) |
| NQO1 | Emphysema (GOLD 3-4), smokers, ex-smokers | No-emphysema, lung cancer patients, never smokers, smokers | ↓ | Lung tissue (whole lung homogenates) | 10 | RT-qPCR | (36) |
|  | COPD (GOLD 0, 1, 2-4), smokers | No-COPD, smokers | ↑ | Epithelial cells obtained by bronchial brushings | 38 | Global gene expression using Affymetrix arrays | (52) |
|  | COPD | No-COPD, smokers, nonsmokers | ↑ | Airway epithelial cells | 80 | mRNA expression profile datasets screening | (58) |
|  | COPD, smokers, ex-smokers | No-COPD, lung cancer patients, smokers | ↑ | AM | 8 | RT-qPCR | (44) |
|  | COPD smokers | COPD ex-smokers | ↑/⎯ | Bronchial epithelium/nasal epithelium, AM, PBMCs | 54 | RT-qPCR | (32) |
|  | No-COPD, smokers | No-COPD, nonsmokers | ↑ | Small airway epithelium | 45 | Microarray analysis, with RT-qPCR confirmation | (69) |
| GPX3 | COPD (GOLD 0, 1, 2-4), smokers | No-COPD, nonsmokers | ↑ | Epithelial cells obtained by bronchial brushings | 38 | Global gene expression using Affymetrix arrays | (52) |
|  | COPD, ex-smokers, smokers | No-COPD, lung cancer patients, never smokers, ex-smokers, smokers | ⎯ | Peripheral lung tissue | 14 | RT-qPCR | (55) |
|  | Severe emphysema, AAT-deficient, smokers, 1 nonsmoker | No-COPD, nonsmokers | ↓ | Peripheral lung tissue | 11 | Microarray analysis | (80) |
| GPX2 | Emphysema (GOLD 3-4), smokers, ex-smokers | No-emphysema, lung cancer patients, never smokers, smokers | ↓ | Lung tissue (whole lung homogenates) | 10 | RT-qPCR | (36) |
|  | COPD | No-COPD, smokers, nonsmokers | ↑ | Airway epithelial cells | 80 | mRNA expression profile datasets screening | (58) |
|  | COPD (GOLD 0, 1, 2-4), smokers | No-COPD, nonsmokers | ↑ | Epithelial cells obtained by bronchial brushings | 38 | Global gene expression using Affymetrix arrays | (52) |
|  | No-COPD, smokers | No-COPD, nonsmokers | ↑/⎯ | Small airway epithelium/AM | 30 | Microarray analysis, with RT-qPCR confirmation | (68) |
|  | No-COPD, smokers | No-COPD, nonsmokers | ↑ | Small airway epithelium | 45 | Microarray analysis, with RT-qPCR confirmation | (69) |
| Keap1 | Emphysema (GOLD 3-4), smokers, ex-smokers | No-emphysema, lung cancer patients, never smokers, smokers | ↑• | Lung tissue (whole lung homogenates), AM | 10 | Western blot, immunohistochemical and laser scanning confocal analysis | (36) |
|  | COPD, smokers, ex-smokers | No-COPD, lung cancer patients, never smokers, smokers | ⎯/⎯ • | Lung resected tissue (bronchial epithelium, AM) | 12 | RT-qPCR/Immunohistochemistry | (44) |
|  | COPD smokers | COPD ex-smokers | ⎯ | Bronchial epithelium, nasal epithelium, AM, PBMCs | 54 | RT-qPCR | (32) |
| DJ-1 | COPD (GOLD 1-2, 3, 4) | No-COPD, lung cancer patients | ↓/↓• | Lung tissue/serum | 180 | Western blotting/ELISA | (48) |
|  | Emphysema | No-emphysema, smokers | ↑/↓• | AT2B cells | 6 | RT-qPCR/Western blotting, densitometric analysis | (98) |
| Bach1 | Emphysema (GOLD 3-4), smokers, ex-smokers | No-emphysema, lung cancer patients, never smokers, smokers | ↑• | Lung tissue (whole lung homogenates), AM | 10 | Western blot, immunohistochemical and laser scanning confocal analysis | (36) |
|  | COPD (GOLD 1, 2, 3, 4), smokers, ex-smokers | No-COPD, smokers, ex-smokers | ↑ | PBLs | 33 | cDNA gene expression microarray analysis | (84) |
| TXNRD1 | COPD (GOLD 0, 1, 2-4), smokers | No-COPD, nonsmokers | ↑ | Epithelial cells obtained by bronchial brushings | 38 | Global gene expression using Affymetrix arrays | (52) |
|  | COPD, smokers, ex-smokers | No-COPD, lung cancer patients, smokers | ↑ | AM | 8 | RT-qPCR | (44) |
|  | COPD | No-COPD, smokers, nonsmokers | ↑ | Airway epithelial cells | 80 | mRNA expression profile datasets screening | (58) |
|  | No-COPD, smokers | No-COPD, nonsmokers | ↑/⎯ | Small airway epithelium/AM | 30 | Microarray analysis, with RT-qPCR confirmation | (68) |
|  | No-COPD, smokers | No-COPD, nonsmokers | ↑ | Small airway epithelium | 45 | Microarray analysis, with RT-qPCR confirmation | (69) |
| SOD1 | COPD, smokers, ex-smokers | No-COPD, lung cancer patients, smokers | ↑ | AM | 8 | RT-qPCR | (44) |
|  | COPD, ex-smokers, smokers | No-COPD, lung cancer patients, never smokers, ex-smokers, smokers | ⎯ | Peripheral lung tissue | 14 | RT-qPCR | (55) |
|  | COPD/Emphysema, smokers, ex-smokers | No-COPD/emphysema, smokers, ex-smokers, never smokers | ↓• | Blood samples-whole blood | 45 | Immunohistochemistry | (99) |
|  | COPD (GOLD 1, 2), smokers | No-COPD, lung cancer patients, smokers, nonsmokers | ⎯ • | Central bronchial epithelium, peripheral bronchiolar epithelium, alveolar epithelium, AM | 22 | Immunohistochemistry | (61) |
| SOD2 | COPD (GOLD 0, 1, 2-4), smokers | No-COPD, smokers | ↑ | Epithelial cells obtained by bronchial brushings | 38 | Global gene expression using Affymetrix arrays | (52) |
|  | COPD, ex-smokers, smokers | No-COPD, lung cancer patients, never smokers, ex-smokers, smokers | ⎯ | Peripheral lung tissue | 14 | RT-qPCR | (55) |
|  | COPD (GOLD 1, 2, 3, 4), smokers, ex-smokers | No-COPD, smokers, ex-smokers | ↑ | PBLs | 33 | cDNA gene expression microarray analysis | (84) |
|  | No-COPD, smokers | No-COPD, nonsmokers | ⎯/↓ | Small airway epithelium/AM | 30 | Microarray analysis, with RT-qPCR confirmation | (68) |
|  | COPD (GOLD 1, 2), smokers | No-COPD, lung cancer patients, smokers, never smokers | ↑• | Central bronchial and alveolar epithelium | 22 | Immunohistochemistry | (61) |
| SOD3 | COPD, ex-smokers, smokers | No-COPD, lung cancer patients, never smokers, ex-smokers, smokers | ⎯ | Peripheral lung tissue | 14 | RT-qPCR | (55) |
|  | Advanced COPD, Emphysema, ex-smokers | No-COPD, smokers, ex-smokers | ↓ | Lung tissue: AT2B cells | 17 | ScRNAseq | (73) |
|  | COPD (GOLD 1, 2, 3, 4), | No-COPD, smokers, never smokers | ↓•/↑• | Alveolar interstitial area/sputum | 12 | Immunohistochemistry/ELISA | (100) |
|  | COPD, smokers | No-COPD, lung cancer patients, smokers, nonsmokers | ↓•/⎯ | Alveolar, bronchiolar, arteriolar walls = lung interstitium/lung tissue | 20 | Immunohistochemistry/RT-qPCR | (62) |
|  | COPD (GOLD 1, 2), smokers | No-COPD, lung cancer patients, smokers, nonsmokers | ⎯ • | Peripheral bronchioles, AM | 22 | Immunohistochemistry | (61) |
| VDR | COPD (GOLD 1-2, 3, 4) | No-COPD, lung cancer patients | ↓• | Lung tissue | 180 | Western blotting | (48) |
|  | COPD, smokers | No-COPD, never smokers | ⎯/↓• | COPD explant lungs, unused donor lungs | 10 | RT-qPCR/Western blotting | (49) |
|  | COPD (GOLD 1-2, 3, 4), smokers, nonsmokers | No-COPD, lung cancer patients, smokers, nonsmokers | ↓• | Lung tissue | 101 | Western blotting | (50) |
| FOXO3 | COPD, ex-smokers, smokers | No-COPD, lung cancer patients, never smokers | ↓• | Peripheral lung tissue, sputum cells (mainly consist of macrophages and neutrophils) | 17 | Immunohistochemistry, immunoblot analysis | (59) |
|  | COPD (GOLD 1,2, 3), smokers, ex-smokers | No-COPD, lung cancer patients nonsmokers, smokers and ex-smokers | ↓ | Lung tissue | 48 | Gene expression microarray profiling with RT-qPCR confirmation | (63) |

↑ up-regulation, ↓ down-regulation, ⎯ no significant difference, • expression measured at a protein level, ….. measurements were performed at baseline and after a mean follow-up of 49.7 ± 6.9 months, COPD chronic obstructive pulmonary disease, AAT-deficient alpha-1 antitrypsin deficient, AM alveolar macrophages, AT2B cells alveolar type 2 bulk cells, PBMCs peripheral blood mononuclear cells, PBLs peripheral blood lymphocytes, RT-qPCR real-time quantitative polymerase chain reaction, ScRNAseq single cell RNA sequencing, ELISA enzyme-linked immunosorbent assay
